# Nonequilibrium Time Reversibility with Maps and Walks

**DOI:** 10.3390/e24010078

**Published:** 2022-01-01

**Authors:** William Graham Hoover, Carol Griswold Hoover, Edward Ronald Smith

**Affiliations:** 1 Ruby Valley Research Institute, 601 Highway Contract 60, Ruby Valley, NV 89833, USA; hooverwilliam@yahoo.com (W.G.H.); hoover1carol@yahoo.com (C.G.H.); 2 Department of Mechanical and Aerospace Engineering, Brunel University London, Uxbridge UB8 3PH, UK

**Keywords:** nonequilibrium simulations, time reversibility, fractals, baker maps, random walks

## Abstract

Time-reversible dynamical simulations of nonequilibrium systems exemplify both Loschmidt’s and Zermélo’s paradoxes. That is, computational time-reversible simulations invariably produce solutions consistent with the *irreversible* Second Law of Thermodynamics (Loschmidt’s) as well as *periodic* in the time (Zermélo’s, illustrating Poincaré recurrence). Understanding these paradoxical aspects of time-reversible systems is enhanced here by studying the simplest pair of such model systems. The first is time-reversible, but nevertheless dissipative and periodic, the piecewise-linear compressible Baker Map. The fractal properties of that two-dimensional map are mirrored by an even simpler example, the one-dimensional random walk, confined to the unit interval. As a further puzzle the two models yield ambiguities in determining the fractals’ information dimensions. These puzzles, including the classical paradoxes, are reviewed and explored here.

## 1. Introduction

Nonequilibrium molecular dynamics evolves according to time-reversible equations of motions which govern the evolution of positions *q* and momenta *p*. However, the time-reversible equations are at odds with observations, namely that entropy increases because the real processes are not reversible. Loschmidt’s paradox is the observation that this irreversible behavior emerges from these systems despite the time-reversible equations describing their motion. The evolution of these systems can be thought of in terms of phase space, a plot of the *p* against *q* coordinates for all 6N dimensions. A position in phase space describes a unique configuration of the system, which changes as the system evolves. After a long enough period of time, the Poincaré recurrence time, theoretically the evolving system must return to its starting position in phase space. As a result, the entropy of a system cannot grow if it is possible for the system to return to its initial conditions in phase space. This is Zermélo’s paradox. Zermélo’s paradox, also known as Zermélo’s recurrence objection or Wiederkehreinwand, was raised as an objection to the validity of Boltzmann’s H-theorem. Simulations with nonequilibrium molecular dynamics show that time-reversible systems generate multi-fractal attractor and repellor pairs in phase space [1,2]. Understanding of these multi-fractal structures sheds light on both Loschmidt’s (reversibility) Paradox and Zermélo’s (recurrence) Paradox. The Baker map is one of the simplest models which can generate a similar fractal phase space, and these fractals can be shown to have similar properties to the fractal dimensions exhibited by continuous Nonequilibrium molecular dynamics flows in three or more dimensions [3,4]. The idea of the present work is to use these maps as an even simpler way of studying the same fractal formation and dissipation (in one and two dimensions). A version of the Baker map which is dissipative and written in terms of (q,p) coordinates is used, as well as a simpler random walk version which can be shown to give identical fractal phase-space attractors.

These maps allow a study of entropy growth by modelling a similar ergodic system. The averages produced by molecular dynamics and Gibbs’ statistical mechanics agree for “ergodic” systems, systems with a dynamics able to access all of the (q,p) coordinate-momentum states included in Gibbs’ statistical averages. Ergodicity is actually a purely theoretical construct for manybody systems. The time required for a nearly complete averaging over a many-dimensional phase space grows exponentially with system size, and exceeds the age of the universe when the number of degrees of freedom is a dozen or so. On the other hand the ergodicity of few-body models can be established and studied. Two hard disks, with periodic boundary conditions is a simple example. The eight-dimensional phase space can be reduced to three (enough for chaos) by imposing symmetry and constant energy on the dynamics. A further simplification can be attained by considering maps, in which the “next” system state is a function of the “current” state of the system. The two simplest such maps are described here, the two-dimensional Baker Map and a closely-related one-dimensional Confined Random Walk. We start by briefly introducing the methodology for these maps in Section 2, for more background the reader is referred to previous work [5,6]. The results and discussion are then presented in the next Section 3, covering results from the two Baker maps including the change in phase volume, Lyapunov growth, irreversibility, Poincaré recurrance, information dimension and parallel implementation of the Baker map. Finally, conclusions are presented in Section 4.

## 2. Methodology

The original Hopf Baker map is discussed first, before explaining the link to time-reversible molecular dynamics, the dissipative form of the Baker map and finally the random walk version which gives similar behaviour.

### 2.1. Hopf’s Equilibrium Baker Map E(x,y)

**Figure 1:**
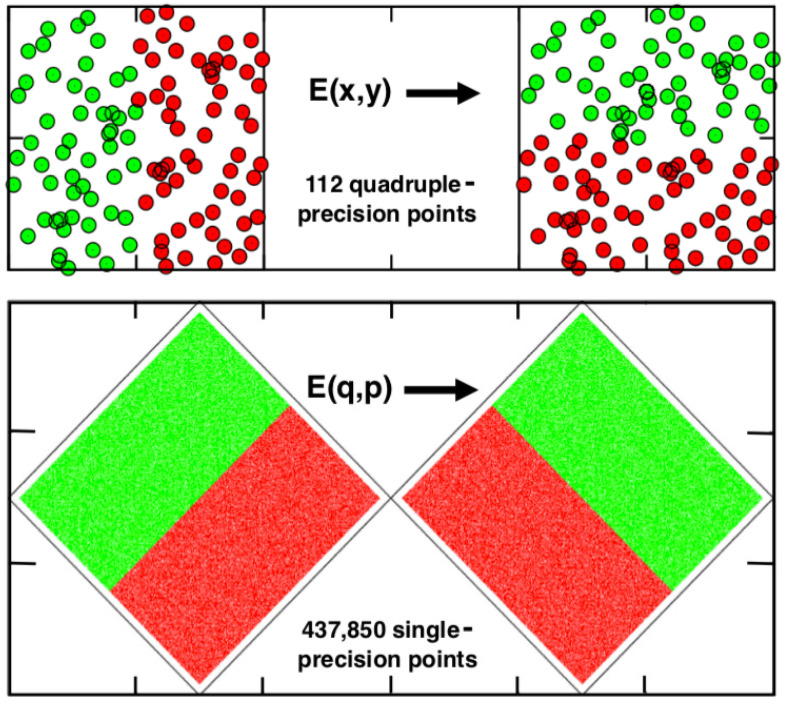
Hopf’s deterministic Baker Map E(x,y) maps the left/right sides of the unit square into the top/bottom halves with each iteration. The continued doubling in the *x* direction eventually reaches a fixed point. The Figure shows a series of 112 points generated with quadruple-precision arithmetic. Evidently Hopf could not imagine a computational implementation of his map! The diamond-shaped lower version of the map, 2×2 rather than a unit square, produces a long periodic orbit and is, unlike Hopf’s, time-reversible.

Nearly a century ago Hopf introduced his Baker Map, reminiscent of a bread baker’s dough-kneading action. We construct it here in the unit square, 0<x,y<1. See Figure 1. The mapping at the top is based on two choices for subsequent points: For a current (green) point with x<1/2 we choose

(1) $$\begin{array}{c} x_{\mathrm{new}}=2x\;\mathrm{and}\;y_{\mathrm{new}}=\left(y+1\right)/2\;, \end{array}$$

while for a current (red) point with x>1/2 we choose instead

(2) $$\begin{array}{c} x_{\mathrm{new}}=2x-1\;\mathrm{and}\;y_{\mathrm{new}}=y/2\;. \end{array}$$

Hopf’s interest was “ergodic theory” and this Baker model can be proved ergodic *most* of the time.

Here “most” of the time means choosing an irrational initial condition. Rational beginnings or numerical finite-precision simulations lead promptly to fixed points instead. The fixed-point mechanism is simply the repeated doubling of the fractional part of the *x* coordinate. In fact Figure 1 shows the typical fate of a numerical implementation of Hopf’s equilibrium map. The quadruple-precision simulation illustrated in the figure generates 112 (x,y) states, of which the last is a fixed point. Single and double-precision simulations likewise come to fixed-point ends, after 23 and 52 iterations of Hopf’s map. If Hopf’s evolution is instead described in terms of a rotated (q,p) coordinate system the result is a relatively long periodic orbit rather than a fixed point. Even single precision gives a period of hundreds of thousands of iterations. See again Figure 1.

In the summer of 1997 at the Eötvös University school/workshop meeting “Chaos and Irreversibility” Hoover and Posch [3] introduced time-reversibility and dissipation into a generalized Baker Map. Tasaki, Gilbert, and Dorfman [4] analyzed families of similar maps at that same meeting. See as well George Kumiĉák’s related work [7] from 2004. Next, we focus on the specific dissipative and time-reversible (q,p) map considered by Hoover and Posch, and described in what follows.

### 2.2. From Equilibrium to Nonequilibrium with Time-Reversible Maps

Molecular dynamics is “time-reversible” if the previous step can be recovered by a three-step process: [1] At time t+dt change the signs of the momenta, {+p→−p}; [2] Propagate the resulting reversed (q,−p) state (backward) to time *t*; [3] Change the signs of the momenta, matching the original (q,+p) state at time *t*. A time-reversible Baker Map B(q,p) would obey the relation $B^{-1}=T\;\times \;B\;\times \;T$, where T is the (time reversal) mapping that reverses the momenta. If we choose (q,p) coordinate-momenta variables in a 2×2 rotated Baker map, the “equilibrium” (incompressible) map E, illustrated in Figure 1 has the analytic form

if(q < p)

qnew = (5/4)q - (3/4)p + 3d

pnew = -(3/4)q + (5/4)p - d

else if(q > p)

qnew = (5/4)q - (3/4)p - 3d

pnew = -(3/4)q + (5/4)p + d

end

**Figure 2:**
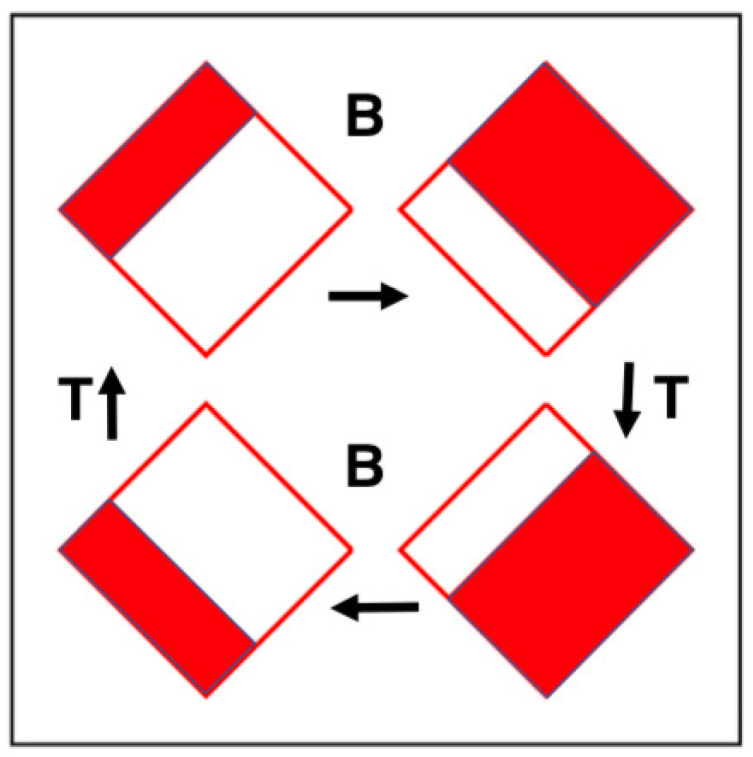
The deterministic Baker Map B doubles an area dqdp in the red region and halves an area in the white. The time-reversal Map T changes the sign of the vertical “momentum-like” variable p. The diamond-shaped domain of the map is $|\;q\pm p\;|<\sqrt{2}$. A counterclockwise circuit of the four states follows if B is replaced by B${}^{-1}$ as T and T${}^{-1}$ are identical.

**Figure 3:**
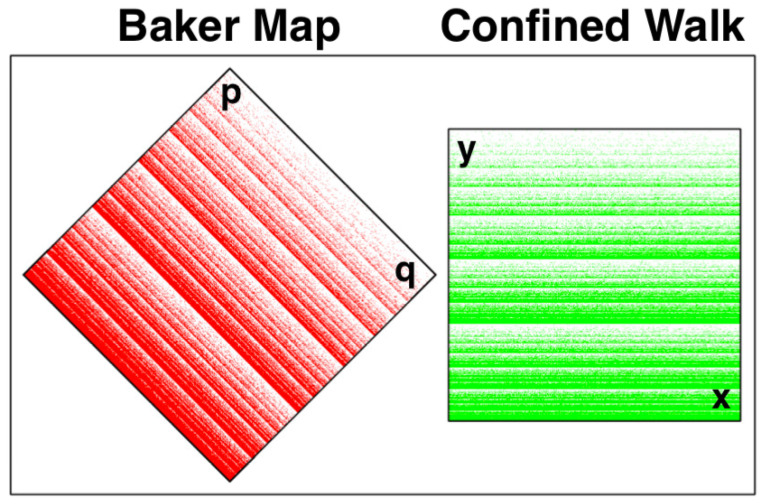
One million iterations of the Baker Map and the Confined Walk are compared. A scaling and translation of the Baker Map solution at the left to the unit square replicates the solution of a stochastic confined random walk problem where *x* and *y* are stochastic variables. The walk confined to the unit interval 0<y<1 is generated with a random number relating the “next” value of y to the “last”. The latter is either y/3 or (1+2y)/3, corresponding to steps to the bottom third or upper two thirds of the green million-iteration solution at the right of Figure 3. The (q,p) Baker Map at the left and the (x,y) Confined Walk at the right provide indistinguishable fractals when rotated 45 degrees and scaled by a factor of two, as shown in the figure. The Confined Walk shown there occupies a unit square, 0<(x,y)<1. We show a 2×2 version, with $|q\pm p|<\sqrt{2}↔\left|x,y\right|<1$, here to clarify the details of the fractal structure.

Where d = sqrt(1/8). This “motion” is analogous to ordinary Hamiltonian mechanics, where the phase volume dqdp is conserved by Hamilton’s motion equations. In *nonequilibrium* molecular dynamics the extraction of heat, corresponding to entropy loss, leads to a continuous loss of phase volume. A mapping analogy can be illustrated by constructing a compressible mapping, as shown in Figure 2 and Figure 3.

Nonequilibrium Molecular Dynamics has a half-century history of providing simulations of viscous and heat-conducting flows driven by boundary differences in velocity and temperature respectively [8,9]. Stationary state simulations are typically stabilized by time-reversible thermostat forces, linear in momentum. These “thermostat forces” impose the desired thermalized boundary conditions [10,11,12] to maintain nonequilibrium steady states. Such simulations are irreversible despite their time-reversible motion equations. These simulations invariably provide the positive viscosities and heat conductivities associated with Loschmidt’s paradox.

Phase-space analyses of small-scale steady-state nonequilibrium simulations indicate stable periodic steady-state structures, “attractors” in phase space. The attraction is termed “strange” because constrictive attractor dynamics simultaneously exhibits expansive Lyapunov instability, with small perturbations growing exponentially in time. Despite this expansive instability the phase volume comoving with a trajectory point is attractive, shrinking with time as the computation settles onto a periodic, but Lyapunov unstable, orbit. This periodicity illustrates Zermélo’s recurrence paradox and implies that the actual dimensionality of computational nonequilibrium steady states is only unity. However, as the computational precision is further refined the orbit lengthens, with the length soon becoming too long to measure and with the one-dimensional trajectory defining a natural measure (or coarse-grained probability density) with a fractal information dimension in the phase space.

Thus precise long-time nonequilibrium trajectories in phase space come to define *fractal* structures, still space-filling, but only sparsely. The dimensionality of these structures is significantly less than that of the equilibrium phase space supporting their nonequilibrium dynamics. The “information dimensions” of these fractals can be estimated by phase-space binning, as we shall demonstrate. In fact such phase-space dimensionality descriptions are not completely clearcut as their description with simple models like Baker’s reveals a sobering nonuniform convergence. The simplest model flows exhibiting these interesting fractal formations are one-particle systems with three-dimensional phase spaces [1,2]. Those spaces include a single coordinate-momentum pair along with a time-reversible friction coefficient ζ. The friction coefficients stabilize nonequilibrium steady states.

In the remainder of this work we discuss the two simplest models, a two-dimensional Baker Map and a related one-dimensional confined random walk. Their study sheds light on the coexistence of time reversibility with the dissipation typifying strange attractor structures in model phase spaces.

### 2.3. The Time-Reversible Dissipative Baker Map

Maps, as opposed to flows, can exhibit similar time-reversible dissipation while occupying only one or two phase-space dimensions. We consider here a compressible version of the “Baker Map” [3,4,7]. It evolves a single coordinate-momentum pair of variables (q,p) as is illustrated in Figure 2. A longtime solution appears at the left in Figure 3. In the two-dimensional (q,p) coordinate-momentum phase space compressible Baker-Map dynamics simply generates a new (q,p) pair from the old one, as described by a linear map. At the top of Figure 2 the smaller red area with $q-p<\sqrt{\left(2/9\right)}$ is expanded twofold by the Baker Map B. The expanding map has the analytic form:

qnew = +(11q/6) - (7p/6) + 14d

pnew = -(7q/6) + (11p/6) - 10d

The constant d is $\sqrt{\left(1/72\right)}$. Notice that the expanding map has a (q,p) Jacobian determinant of (121−49)/36=2, signalling a doubling of area with each iteration. In the larger white region the map, likewise linear, contracts:

qnew = +(11q/12) - (7p/12) - 7d

pnew = -(7q/12) + (11p/12) - d

Here the determinant of the contracting map is (121−49)/144=1/2 signifying twofold compression. Figure 3 illustrates a million-iteration solution of the mapping equations.

Figure 2 illustrates the time-reversibility of the map. First, starting at the upper right of the figure, change the sign of the vertical “momentum variable” *p* with the map “T”, ending at lower right; next map forward with “B” to lower left; last reverse time again with “T” returning (q,p) to its original top left pre-mapped location, demonstrating that the inverse mapping is B${}^{-1}$ = TBT. This identity defines a time-reversible mapping.

Though the map looks even-handed compression invariably wins out over expansion. It must! A little reflection identifies compression with stability and expansion with its opposite, instability. An unphysical hypothetical system in which expansion wins out over compression would correspond to numerical instability with an exponential divergence of the comoving phase volume. In numerical work only about ten percent of the simple Baker-Map iterations are computationally reversible in the sense that applying the inverse mapping undoes the most recent iteration precisely so that B${}^{-1}$B leaves (q,p) unchanged.

### 2.4. Random Walk Analog of the Baker Map

A random-walk analog for the progress of the Cartesian *y* variable on the unit interval can be modelled given current values of x and y. A new value xnew can be chosen at random, while ynew depends upon both a random number r where 0<r<1 and the current value of y:

if(r.lt.1/3)

ynew = (1+2y)/3

else if(r.gt.1/3)

ynew = y/3

end

The righthand side of Figure 3 illustrates the strange-attractor character of numerical solutions of this confined walk problem. The Confined Walk fractal at the right is related to the Baker Map fractal at the left by a 45-degree counterlockwise rotation. Evidently, due to the exponential growth in the walk’s horizontal *x* direction that distribution is random and can be modelled by a good random-number generator. In the *y* direction the distribution is a self-similar fractal, repeating in an infinite set of bands with each band smaller than its predecessor by a factor of (2/3) and including one-third as many points.

## 3. Results and Discussion

### 3.1. Irreversibility through Shrinking Phase Volume

An understanding of the shrinking phase volume, which leads to an apparent fractal (fractional dimensional) phase-space object is straightforward in the Baker Map example of Figure 2. The map, whether in the red region or the white, always expands in the northwest-southeast direction parallel to lines of constant q+p. This expansion characterizes Lyapunov instability, the growth of microscopic perturbations to macroscopic scale. In the perpendicular direction, parallel to lines of constant q−p, the map contracts to the self-similar fractal structure displayed in Figure 3.

#### 3.1.1. Lyapunov Instability and Exponential Growth

**Figure 4:**
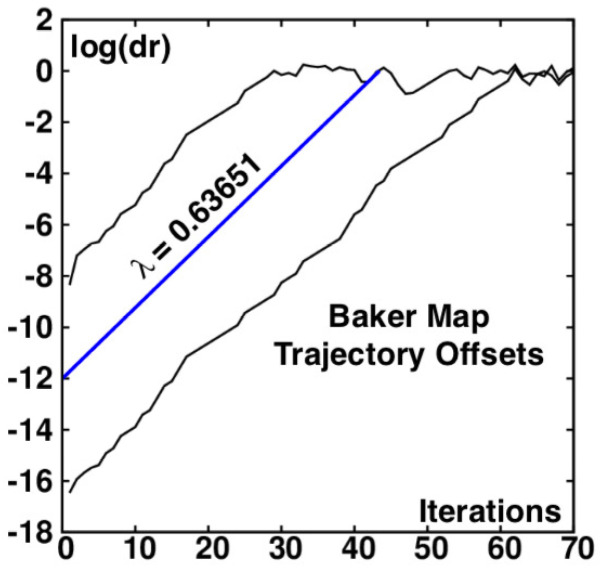
Comparison of the differences $dr=\sqrt{dq^{2}+dp^{2}}$ between single and double precision simulations (above) of the Baker Map and double and quadruple precision simulations (below) with all three trajectories started at the origin. The straight blue line has a slope corresponding to the largest Lyapunov exponent, $\lambda_{1}=0.63651$.

Figure 4 illustrates the exponential character of the expansive northwest-southeast growth by displaying the offset between single- and double-precision simulations, the uppermost of the three curves in the figure. Both simulation types begin at the (q,p)=(0,0) origin at the center of the diamonds shown in Figure 1, Figure 2 and Figure 3. The initial separation immediately reflects the roundoff error of the single-precision mapping, of order $10^{-10}$. The single-double separation increases by ten orders of magnitude in about 40 iterations of the map. Similarly, the double-quadruple separation likewise grows exponentially. In that more-nearly-accurate more-precise case the growth rate is the same $\;e^{+\lambda_{1}t}$. $\lambda_{1}$ is the largest Lyapunov exponent. Its numerical value is 0.63651. The exponent and the blue middle line in Figure 4 drawn with its slope corresponds to averaging the growth rates in the red and white regions, ln(3) and ln(3/2) respectively, taking into account that the white compressive region is twice as likely as the red. The result is the time-averaged expansion rate:

(3) $$\begin{array}{c} \langle \;\lambda_{1}\;\rangle =\left(1/3\right)\ln\left(3\right)+\left(2/3\right)\ln\left(3/2\right)=\left(1/3\right)\ln\left(27/4\right)=0.63651\;. \end{array}$$

Similarly, the compression perpendicular to the expansion gives the second Lyapunov exponent:

(4) $$\begin{array}{c} \langle \;\lambda_{2}\;\rangle =\left(1/3\right)\ln\left(2/3\right)+\left(2/3\right)\ln\left(1/3\right)=\left(1/3\right)\ln\left(2/27\right)=-0.86756\;. \end{array}$$

This analysis is in good agreement with the numerical data. With an initial double-precision roundoff error of order $10^{-17}$ the exponential loss of accuracy expands to unity after about 60 iterations of the map: $10^{+17}≃e^{0.63651\times 60}$, as in the lower curve.

#### 3.1.2. Compression with Expansion Leads to Irreversibility

Despite the exponential growth of northwest-southeast separations the overall phase volume shrinks. On average a white area is halved two thirds of the time while a red area doubles one third of the time. Thus overall the comoving area decreases as $2^{\left(-t/3\right)}$ with *t* iterations. That area is soon reduced to a vanishingly-small fraction of its initial value. That fraction is of order $e^{-100}$ for a thousand iterations of the map. This contraction of area accounts for the sparse appearance of the thousands of mapped points in the smallest-scaled regions of Figure 3. The mean densities in the self-similar bands decrease with increasing values of *y*:

(5) $$\begin{array}{c} \langle \;\rho \left(0<y<\frac{1}{3}\right)\;\rangle =2^{1}\;;\langle \;\rho \left(\frac{3}{9}<y<\frac{5}{9}\right)\;\rangle =2^{0}\;;\langle \;\rho \left(\frac{15}{27}<y<\frac{19}{27}\right)\;\rangle =2^{-1}\ldots \;. \end{array}$$

**Figure 5:**
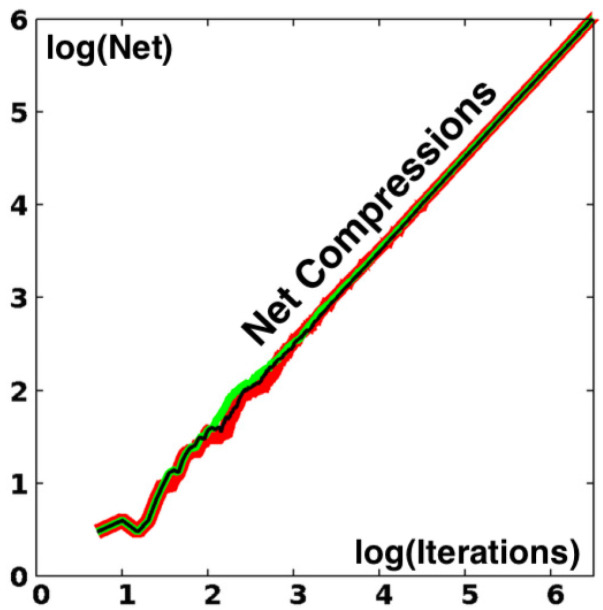
Excess of compressive over expansive iterations of the single-(red), double-(green), and quadruple-(black) precision Baker maps. The differences between them are visible between 20 and 1000 iterations of the maps. The final values of the excess are 1,000,742, 1,000,250, and 998,236 for the three sets of three million iterations beginning at (0,0).

The expansive “strange” portion of the mapping is responsible for this decrease in density even though it is unlikely relative to compression. Consider an initial point at the origin (q,p)=(0,0) and follow it forward in time, counting the net number of compression steps. Figure 5 shows the results of three million iterations of the map in single, double, and quadruple precision. As expected the net numbers of compressive iterations are “close to” [within a thousand or so] one-third the total.

### 3.2. Poincaré Recurrence of the Baker Maps

A fringe benefit of the diamond-shaped Baker Map is the relatively long Poincaré recurrence time. The computational “noise” contributing to this longevity can be traced to the irrational square roots in the mapping. The single-precision mapping which we have used in Figure 4 and Figure 5 has a periodic orbit of length just over a million iterations, 1,042,249 to be precise. The double-precision mapping settles into a periodic orbit repeating after a few trillion iterations [5]. The (x,y) Cartesian coordinate version of the map corresponding to the orientation at the right in Figure 3 is not well suited to computation due to its many short periodic orbits, many of which are stable (which we view as “unphysical”. A clear and comprehensive analysis of the generalized Baker Map problem, with arbitrarily small and large expansions and contractions, was presented by Kumiĉák in 2005 [7]).

### 3.3. Characterizing Chaos in the Baker Map

**Figure 6:**
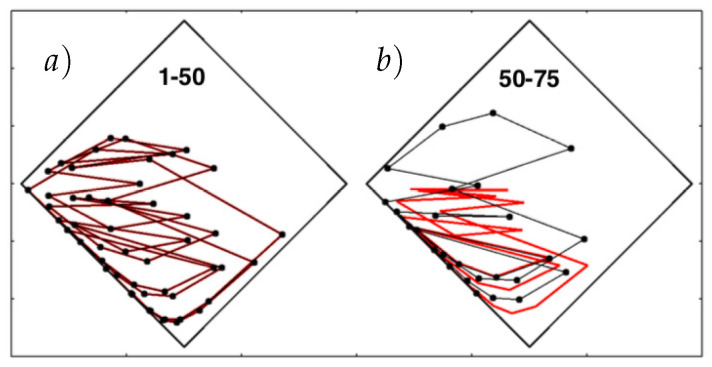
Additional iterations, (**a**) 1 to 50 and (**b**) 50 to 75 in double (black) and quadruple (red) precision for the two-dimensional (q,p) Baker Map B with the initial point at the origin (0,0). Lyapunov instability makes the difference between the two solutions visible after about 70 iterations, as can be seen in (**b**).

Numerical solutions carry a fixed number of digits, 9, 17, and 36 for single-, double-, and quadruple-precision numbers on the unit interval. Chaos is characterized by the exponential growth of small perturbations. The Baker Map’s largest Lyapunov exponent is 0.63651, so that the differences seeded by roundoff between single- and double-precision, and between double- and quadruple-precision solutions can be estimated from Figure 4 just as easily as from the analytic growth rates. That figure illustrates the offsets and Figure 6 the trajectory differences between 50 and 75 iterations of the double and quadruple-precision maps. At 50 iterations, corresponding to multiplying by $e^{32}≃10^{14}$, roundoff error has not yet amplified the difference between double and quadruple precision to visibility, while 75 iterations are more than sufficient to lose any visible correlation between the two solutions.

The right side of Figure 3 shows that a stochastic view of the map, traceable to its chaos, is quite proper. Before the mapping shown at the top of Figure 2 is executed we note that the white southeastern area is twice that of the northwestern red area so that compressive steps (into the highest-density lower third of the unit square) are twice as likely as expansive steps (into the upper two-thirds). The preponderance of the excess, lower–upper, is shown in Figure 5 and approaches, on average, t/3 as the number of iterations *t* increases. We would expect the error in this statistical estimate to become visually negligible, say one percent, once the number of iterations is of order $10^{4}$. A look at the figure shows that the statistical bumpiness away from a straight line becomes visually negligible between 1000 and 10,000 iterations, just as one would expect for a stochastic, rather than deterministic, process.

**Figure 7:**
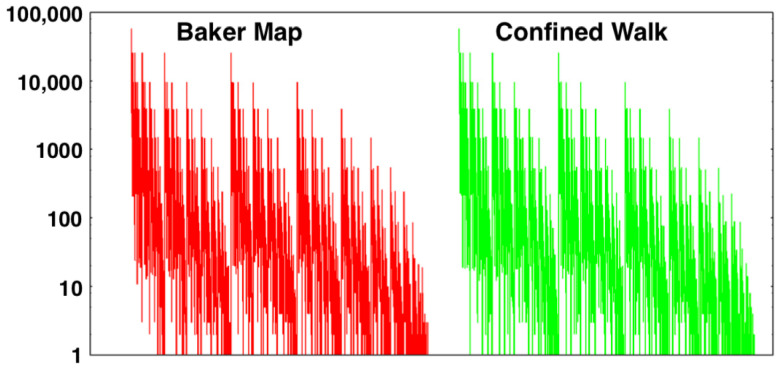
Comparison of the *y* coordinate distribution of the Baker Map in the unit square converted from (q,p) with the Confined Walk distribution obtained using the FORTRAN random-number generator random${}_{-}$number(r). The Map and Walk data, one million points for each, have been collected here and displayed in $2187=3^{7}$ bins of width $3^{-7}$.

It is an article of faith that the *x* motion of the (x,y) map is completely random [13]. This statistical view is consistent with our numerical work so that we believe it is fully justified. It is easily checked by comparing bin populations for the Map and the Walk after millions or billions or trillions of iterations. See Figure 7 for a million-iteration sampling of 2187 bins for both approaches. Let us consider the confined random walk problem in more detail next [5,6,13].

### 3.4. Results from the Random Walk Baker Map

**Figure 8:**
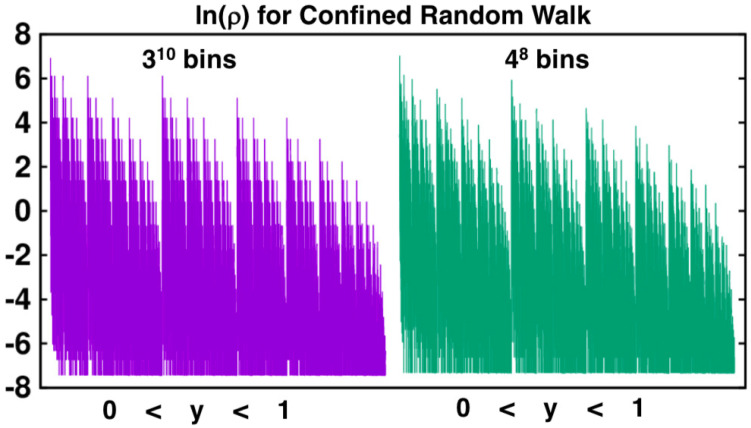
Fractal probability densities for confined walks with bin sizes $\delta =3^{-10}$ and $4^{-8}$. For both bin sizes $\int_{0}^{1}\rho \left(y\right)dy=1.$ For $10^{8}$ iterations of the map were used for the point set that was analyzed with both these choices of binning.

Figure 3 and Figure 7 compare the distributions from one million iterations of the two-dimensional Baker Map with those from the same number of iterations applied to the confined walk. In Figure 7 the unit-interval *y* values have been “binned” into $3^{7}=2187$ bins of equal width, δ=1/2187. Initially the doubling of density with steps to the bottom third of the square and halving with steps to the upper two thirds gives rise to probability density steps of factors of four. Later, in the steady state and visible in Figure 8 where the bin probabilities are plotted on a logarithmic scale spanning 16 e-foldings, the regular steps stand out in the lefthand 3-based binnings but are less distinct in the righthand 4-based ones. This difference, along with the continual increase in e-foldings with iterations, suggests the possibility of convergence difficulties in characterizing the resultant fractal.

The only singularity in the linear Baker Map is the border line separating the red and white regions in Figure 2:

(6) $$\begin{array}{c} q-p=-\sqrt{\left(2/9\right)}\;\mathrm{Baker}\;\mathrm{Map}\;⟷x=\left(1/3\right)\;\mathrm{Random}\;\mathrm{Walk} \end{array}$$

Evidently this measure-zero set of singular points is enough to generate the everywhere-singular fractal distribution of the analytic “{y}” and the computational “{y}”. Let us consider further details of the latter set next.

### 3.5. Nonuniform Convergence of the Information Dimensions

The definition of the information dimension $D_{I}\left(\delta \right)$ for a set of points describes the dependence of the probability density of points on the size of the sampling bins, δ. Although other fractal dimensions can and have been defined and studied, the information dimension is uniquely significant. Unlike the correlation dimension $D_{I}$ is unchanged by simple coordinate transformations [14].

**Figure 9:**
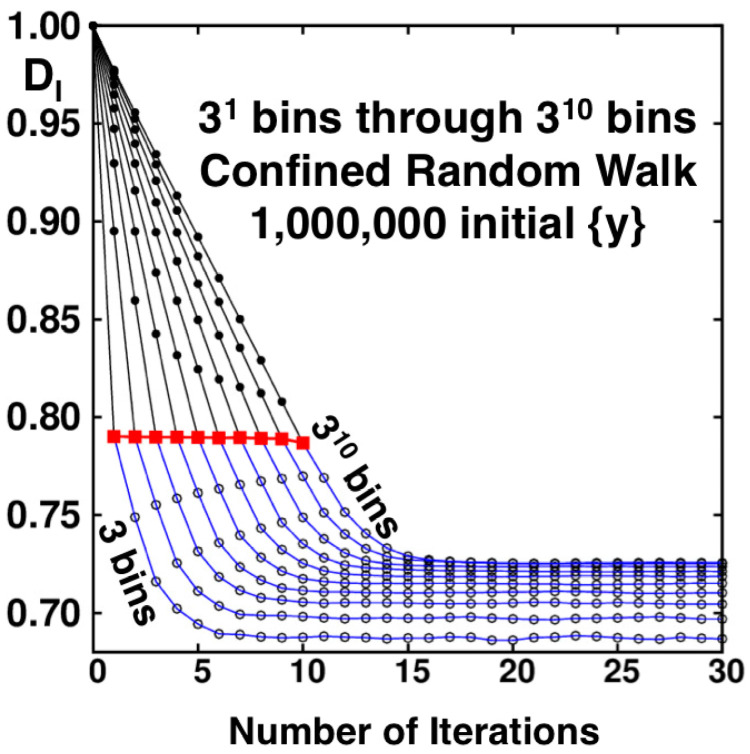
The information dimensions $D_{I}$ of the developing random-walk fractal as ten functions of the number of iterations. The number of bins characterizing each curve varies from $3^{1}$ to $3^{10}$ for each of thirty iterations. Each point corresponds to an averaged $D_{I}$ from one million equally-spaced initial conditions on the unit interval 0<y<1. In the limiting special cases (shown as red squares) that the number of iterations is equal to the logarithm, base-3, of the number of bins, the information dimension follows from Farmer’s analysis [5], [(2/3)ln(2/3)+(1/3)ln(1/6)]/ln(1/3)=0.78967. When the number of iterations approaches infinity ahead of the number of bins, the dimensionality is substantially lower, $D_{I}≃0.741_{5}$ rather than the Kaplan-Yorke conjectured value (based on the Baker-Map Lyapunov exponents) 0.7337.

To seek uniqueness both the number of bins and the number of points per bin must approach infinity in averaging the bin probabilities: $D_{I}=\langle \;\ln\left(\mathrm{prob}\right)\;\rangle /\ln\left(\delta \right)$. To visualize taking this limit we illustrate the result of iterating a set of one million equally-spaced initial points on confined random walks: $0<\{y_{i}\left(t\right)\}<1$. We explore thirty iterations: 0<t<31. The confined random-walk iterations are governed by the output of the FORTRAN random number generator random${}_{-}$number(r). The dimensionality data are analyzed here using $3^{n}$ bins, with *n* varying from 0 to 10. The finest grid has $3^{10}=59,049$ bins of equal width δ=1/59049. By combining the contents of 3, or 9, or 27, or…contiguous bins the entire set of 30 stepwise information dimensions for the ten binning choices can be obtained from a single run. The apparent information dimensions for the 300 problems (thirty iterations with ten bin sizes) are plotted as the ten lines shown in Figure 9.

**Figure 10:**
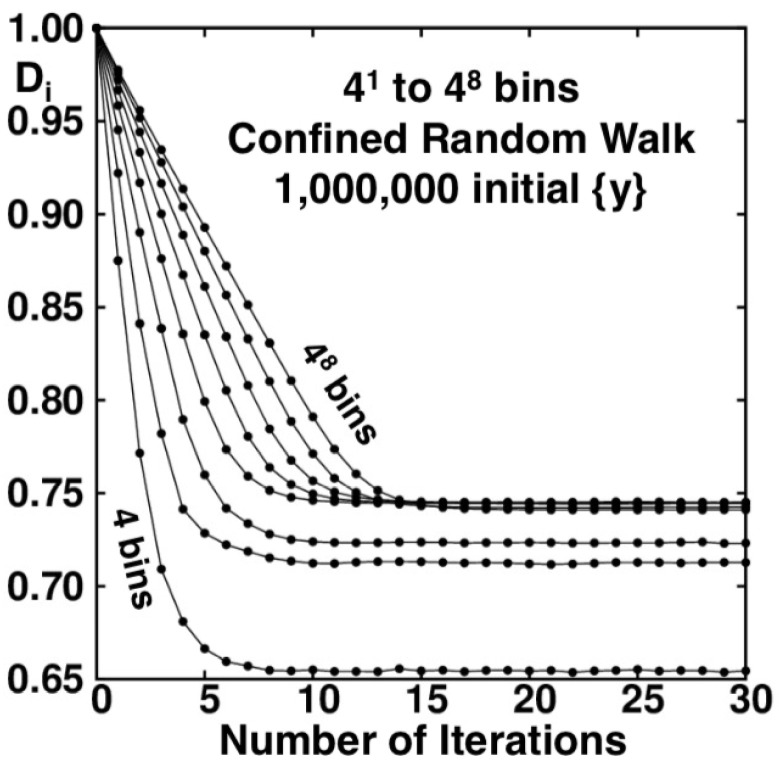
The information dimension $D_{I}$ of the developing random-walk fractal as a function of the numbers of bins and iterations. The number of bins varies from $4^{1}$ to $4^{8}$ for thirty iterations. As in Figure 7 each point corresponds to $D_{I}$ for one of the ten samples of one million initial conditions.

The Baker-Map function, $y=[\left(q+p\right)/\sqrt{2}+1]/2$ provides the same fractal as does the confined walk, penetrating, in both cases, to a scale smaller by a factor 3 with each iteration. For this reason powers of (1/3) are the “natural” bin sizes for analyzing the Baker-Map function [5,13] and the confined walk 0<y<1. Although reciprocal bin widths which are powers of 3 are “natural” for the Baker Map and its confined walk analog, an embarrassing variety of choices is possible. As an example bin widths which are the first eight powers of 4 (a subset of bin widths which are powers of 2) provide the information dimension estimates shown in Figure 10. Similar behaviour and convergence is observed in the case of 1/2, 1/4, 1/5 and 1/7 [15], so the 1/4 case is chosen as representative in the comparison to the 1/3 case. For additional examples see Reference [14]. The totality of these results is paradoxical because they indicate a limiting information dimension of $0.741_{5}$ from the series of widths ${\left(1/3\right)}^{n}$ and a *different* limit, 0.7337, from the series of widths ${\left(1/4\right)}^{n}$. This difference suggests a persistent difference of distributions in the limiting case(s) δ→0. This nonuniform convergence caught us completely by surprise.

The lines in both Figure 9 and Figure 10 appear to plateau to different fixed value depending on bin resolution. This is a consequence of the increased ability to resolve the phase space fractals as number of bins increases. For increasing numbers of bins, with enough iterations the plateau values becomes similar but the actual convergence is very slow, only appearing on logarithmic axes.

The dependence of the limiting information dimension on the bin-width power law, giving either $0.741_{5}$ or 0.7337, suggests a look at the distributions themselves. As the limiting case(s) are singular everywhere, we arbitrarily choose to compare probability densities for both $3^{10}$ and $4^{8}$ bins in Figure 8. Both simulations include exactly the same set of 100,000,000 iterations. The density steps with $3^{10}$ bins are markedly sharper than those with $4^{8}$ and the details of the boundaries between vertical strips are likewise better defined for the finer (65,536 rather than 59,049 bins) mesh.

**Figure 11:**
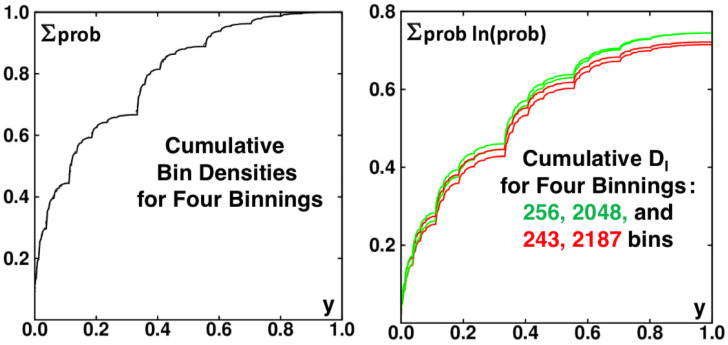
Cumulative densities and information dimensions are compared for bin widths (from bottom to top at the right) of ${\left(1/3\right)}^{5},\;{\left(1/3\right)}^{7},\;{\left(1/2\right)}^{11},\;{\left(1/2\right)}^{8}$. The information dimensions for much narrower bins suggest different limiting dimensionalities for the two bin families.

A clearer picture of the binning dependence follows from the cumulative distributions of density and information, shown for the same data used in Figure 8. Figure 11 shows both the density and the information dimension as cumulative sums for two pairs of similar binnings, ${\left(1/3\right)}^{5}∼{\left(1/2\right)}^{8}$ and ${\left(1/3\right)}^{7}∼{\left(1/2\right)}^{11}$. Because the underlying data are identical the densities always agree, within one bin width. The information dimensions are quite different with powers of 2 both giving $D_{I}=0.745$, significantly greater than $D_{I}=0.715$ and 0.725 for the “natural choice" of powers of three, 243 and 2187 bins. Numerical work suggests that the difference persists even to infinitesimal bin widths.

### 3.6. Massively-Parallel Implementations of the Confined Walk

The Baker Map and Confined Walk problems appear to be ideally suited to parallel computation. Both these information-dimension problems are “embarrassingly parallel”. This means that during computation there is no need for communication between parallel processors. For both problems ergodicity implies that the long-time-averaged bin-dependent information dimensions,

(7) $$\begin{array}{c} D_{I}\left(\delta \right)=\sum_{\mathrm{bins}}p\ln\left(p\right)/\ln\left(\delta \right)=\langle \;\ln\left(p\right)\;\rangle /\ln\left(\delta \right)\;\left[\;\mathrm{Map}\;\mathrm{or}\;\mathrm{Walk}\;\right]\;, \end{array}$$

are independent of the initial condition. The motivation for the current work was, in fact, our desire to apply a parallel computer to these problems. As the Map and Walk problems are equivalent in their fractal natures we will detail only the simpler case, the confined random walk.

Consider a parallel computer with *N* processors, each with its own separate memory storing an array of bin occupation numbers. Such a machine would reduce the computation time for a given number of iterations by a factor of *N*, provided the *N* initial conditions contain no duplicates and the streams of random numbers are unique on each processor. This optimal *N*-fold “speedup” would not apply to problems requiring the alternative “shared-memory” approach. Sharing a single memory, common to all processors, degrades performance dramatically as the *N* processors compete for access to the global storage. To take advantage of a parallel machine, it is necessary to note that *N* independent sets of bin populations must be separately stored, only to be combined at the completion of all the processors’ iterations.

**Figure 12:**
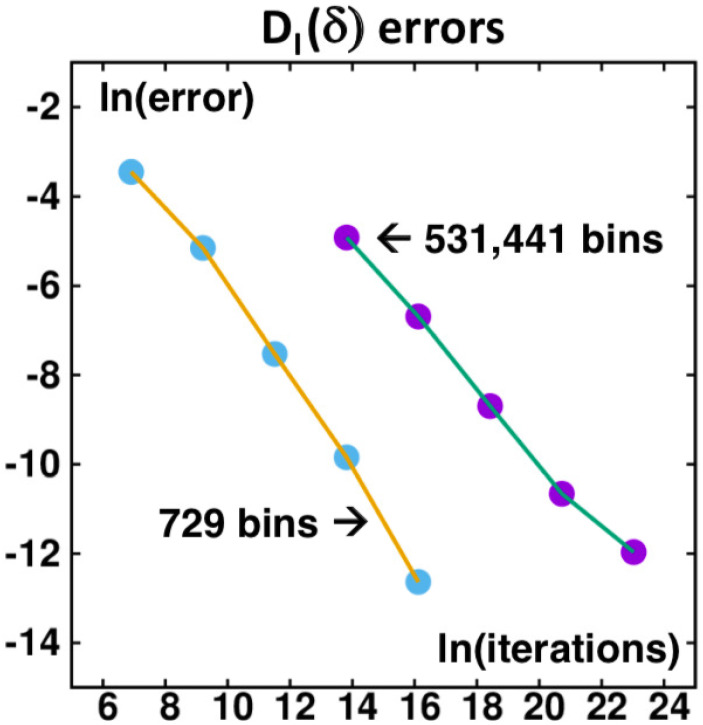
The logarithms of the errors in $D_{I}\left(\delta \right)$ (due to insufficient iterations) are shown for two different bin widths, $\delta =3^{-6}=1/729$ at the left and $\delta =3^{-12}=$ 1/531,441 at the right. For both cases the slope is roughly −1 when plotted as a function of Logarithms of the number of iterations. Thus the error for $D_{I}$ varies inversely with the number of total iterations included. The data plotted were obtained from 100 successive samplings, corresponding to a parallel computation with 100 independent processors. The leftmost and rightmost points correspond to 1000 and $10^{10}$ iterations: ln(1000)=6.908 and $\ln(10^{10})=23.026$.

Let us consider a numerical example to clarify the potential improvement in statistics of a parallel implementation. We imagine a parallel machine with 100 processors applied to the $729=3^{6}$ bin problem. To determine an accurate answer to this problem we begin by carrying out a single serial calculation of 50,000,000,000 iterations. This provides an information dimension of $D_{I}\left(\delta =1/729\right)=0.71858_{2}$. The uncertainty in the last digit is 0.000001 or 0.000002. Next, we consider 100 serial computations (mocking up a 100-processor solution) of 1000, 10,000, and 100,000 iterations each, finding errors in $D_{I}$ of 0.0322, 0.0048, and 0.0005. Further numerical exploration shows that a plot of ln(error) as a function of ln(iterations) has a straight-line slope near −1. Evidently for runs of reasonable length the error varies inversely as the number of iterations. Figure 12 shows errors for both the 729-bin case and the 531,441 $=3^{12}$-bin case suggesting that the inverse relationship is a good “rule of thumb”. For a slowly varying cumulative average twice the final value less that at the halfway point is a reasonable best-guess choice for $D_{I}\left(\delta \right)$.

**Figure 13:**
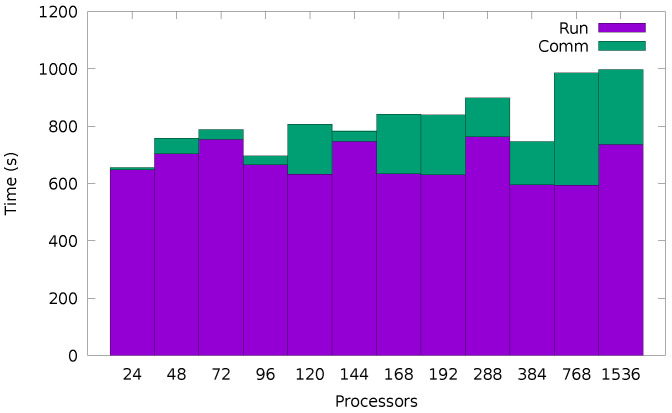
Timing for $10^{10}$ iterations of the Confined Walk using $3^{18}$ bins run on varying numbers of processors. The time is split into the Confined Walk calculation itself (Run), a process which requires no communication between processes, and a communication step (Comm) where the collected bins are accumulated on the root process before being used to calculate $D_{I}$.

It is therefore clear that a parallel implementation can be used to improve statistics. The result of scaling up to 1536 processors is shown in Figure 13 for a Confined Walk run over $10^{10}$ iterations using $3^{18}$ bins. The run time, approximately 600 s, is the calculation of the walk itself. This is seen to be independent of the number of processes, as expected from the embarrassingly parallel nature of the problem. The communication time covers the summing of the value in each bin on each processor to a master set of $3^{18}$ bins on the root processor. The root processor then calculates the value of $D_{I}$ from the set of bins which will contain $N\times 10^{10}$ samples. This communication occurs only once at the end of the run, so despite taking more time as the number of processes *N* grows, it would still be expected to be an insignificant part of a longer run time (e.g., a typical run of 48 h would spend less than 0.1% on communication with 1536 processors).

However, the information dimension requires a process of increasing bin resolution, which necessitates the number of bins be increased toward infinity. As a result, the problem of finding the information dimension quickly becomes memory limited. In the example given here, the 1536 processors are distributed on 24 core “nodes” (essentially a networked computer with a 24 core Central Processing Unit or CPU), each of which has 100 GB (GigaBytes) of random access memory ($10^{11}$ bytes of Random-Access Memory or RAM). The bins’ contents must be stored on each processor. This allows them to run in parallel, which limits the number of bins to no more than  4 GB per processor. The choice here is of $3^{18}$ bins ≈ 1.4 GB, where the count in each bin is stored as a 4 byte integer and is within the limit of 100 GB of random access memory available for the 24 cores. The next power of three, $3^{19}$, would result in storage requirements greater than 4 GB per process. As a result, the supercomputer has the potential to speed up the simulation but only for systems with modest memory requirements. Generally smaller numbers of bins converge rapidly, so compute time are not the limiting step. As a result, this “memory-limited” problem is not ideally suited for acceleration on a supercomputer.

A different approach would be needed if supercomputer speedup is to be possible when collecting the information dimension for the case where number of bins tends to infinity, for example using a dynamic data structure for binning (e.g., a binary tree) or some other way of calculating the information dimension which does not require the allocation of prohibitively large binning arrays. An alternative measure of the fractal dimension could also provide a way forward.

## 4. Conclusions

The finding that nonequilibrium steady states with time-reversible motion equations generate repellor-attractor pairs in phase space has been explored here for two simple models, the Confined Walk and the Baker Map. These models, with one- and two-dimensional phase spaces, are simpler than the continuous flows illustrating chaos [1,2]. The two model systems reveal the details of the singular loss of phase volume resulting in these interesting fractal distributions. Flows, with differential equations rather than maps, require three phase-space dimensions for chaos.

The maps have shown us that nonequilibrium states are rare, occupying only fractional dimensional portions of phase space. Although the time-symmetry of the motion equations guarantees that there is a mirror-image p→−p solution of the motion equations our investigation shows that the dissipative states form an attractor, with a negative Lyapunov-exponent sum. The attractor is a fractional-dimensional stable sink relative to the two-dimensional equilibrium phase space. The paradoxical time-reversed repellor states have positive Lyapunov-exponent sums and are wholly unstable and irreversible. The fractal nature of the non-equilibrium attractors means that their entropies are minus infinity. That property is fundamental to the resolution of the Paradoxes mentioned in the abstract.

The structures of nonequilibrium steady-state phase space flows are qualitatively different to those of Gibbs’ equilibrium ensembles. The steady state flows are directed *from* repellors *to* attractors. The barrier to reversal is exponential. Loschmidt’s reversed states are nearly unobservable, like the attractor states so rare that they never turn up for long. Zermélo’s recurring states are simply typical non-paradoxical dissipative states on stable attractors occupying a fractional-dimensional zero-volume portions of their phase spaces. Thus nonequilibium systems are qualitatively different to Gibbs’ space-filling distributions of points. It is interesting to note that the nonequilibrium fractals have the form of periodic orbits which cannot be reversed. Even for the simple Baker Map only about ten percent of the timesteps can be reversed precisely. The Lyapunov instability going backward in time changes sign, from attractive to repulsive with the Lyapunov instability offset by convergence going forward but completely uncontrolled backward despite the time-reversibility of the equations of motion.

Detailed characterizations of the fractals found in the Walk and Map problems have revealed an unsettling nonuniformity of convergence. Different approaches to the information dimensions of the models [$\delta ={\left(1/3\right)}^{n}$ and $\delta ={\left(1/4\right)}^{n}$, for instance] give different results [5]. However, use of a supercomputer to probe this convergence is found to be limited by the high memory requirements as we increase numbers of bins. The memory bound nature of the problem suggests an alternative approach is needed to reach the limit of zero bin size.

A surprising finding from the one and two-dimensional maps is that the information dimension of these simplest-possible fractals appears to be ill-defined, illustrating the desirability of further work on these fractals. There are other illustrations of the failure of the Kaplan-Yorke conjectured connection of the Lyapunov exponents to the information dimension. Reference [14] is key as to why information dimension is the right property to investigate, as the others are sensitive to minor coordinate-system changes.

We believe that further study of these fractals is warranted. The code to simulate these problems serially, using shared memory and on a supercomputer are provided to facilitate this study. In addition, the 2021 Snook Prize problem seeks to shed light on the information dimension of the fractals, 1.7337 vs. $1.741_{5}$ for the Baker Map, equivalent to 0.7337 vs. $0.741_{5}$ for the Confined Walk problem. Straightforward binning calculations, along the lines of Figure 9 and Figure 10, give histogram probabilities {Prob(δ)} which can then be analyzed for a bin-width dependent information dimension:

(8) $$\begin{array}{c} D_{I}\left(\delta \right)=\langle \;\ln\left(\mathrm{Prob}\left[\delta \right]\right)\;\rangle /\ln\left(\delta \right)\;. \end{array}$$

Computations of $D_{I}\left(\delta \right)$ for small bin sizes appear to lead to three different estimates for the δ→0 limiting “information dimension”! Evidently *the* information dimension [5,6,13,14] of these maps (in the limit that the bin-width δ vanishes) is ill-defined, an interesting example of nonuniform convergence.

## Data Availability

The serial codes, their shared memory (OpenMP) and distributed memory (MPI) version are all included in an open-source repository (http://www.github.com/edwardsmith999/fractal_phase_space (accessed on 31 December 2021). A DOI https://doi.org/10.5281/zenodo.5812056 (accessed on 31 December 2021), has been prepared which is a snapshot of the Github link at the point of submission of this manuscript.
